# Human Magnetism

**Published:** 1845-04

**Authors:** 


I. HUMAN MAGNETISM; ITS CLAIMS TO DISPASSIONATE INQUIRY; BEING AN ATTEMPT TO SHEW THE UTILITY OF ITS APPLICATION FOR THE RELIEF OF HUMAN SUFFERING. By *W. Newnham*, Esq. M.R.S.L., Author of "The Reciprocal Influence of Body and Mind," &c. 8vo. pp. 432. Churchill, London, 1845.

II. MEDICAL REPORT OF THE CASE OF MISS H—— M——. By *T. M. Greenhow*, F.R.C.S. Highley, London, 1845.

III. VITAL MAGNETISM; A REMEDY. By the *Rev. Thomas Pyne*, A.M. Incumbent of Hook, Surrey. 12mo. pp. 80. Highley, London, 1845.

BEFORE we had received Mr. Newnham's volume for review, we had been led by the remarks of some friends to expect that at length there had appeared a calm and temperately-written work on a subject, which has certainly—we shall not at present say how justly—met with rather rough handling from medical critics. On reading the title-page, we augured well; as it seemed to promise what we have for some time past been wishing to meet with, and we thought within ourselves, 'now surely we shall have some facts to deal with, and authentic reports of well-observed cases drawn up by an enlightened and discriminating practitioner, in place of those silly gossiping tales, better suited for the wonderment of an old-maid's tea-party than for discussion by men of reflection and experience, that have of late years been related by the Rev. this or Rev. that, and certified by this literary lady and that learned lawyer.' Our mind, although any thing but favourable to the claims of Mesmerism, was quite prepared to give a patient hearing to whatever might rationally be adduced in its defence, and we were ready to weigh, in the scales of fairness and truth (we trust), any well-authenticated data that might be brought forward by a professional man; the evidence of the *laity* in such a matter being, we need not say, almost entirely valueless.

Such was the state of our feelings when we cast our eyes over the dedicatory epistle, which Mr. Newnham has addressed to his friend the Reverend Thomas Osmond Fry. One expression in it certainly startled us not a little, and somewhat disconcerted the estimate which we had formed of our author's judgment. We proceeded to the perusal of the introductory Chapter, in the hope of recovering our good opinion of him upon better acquaintance; but in this we were disappointed. On, however, we pushed, the mild angel, Charity, "that thinketh no evil," leading the way; but even her sweet influence could not prevail; our fears increased, our hopes gave way, one by one; we became puzzled, perplexed, surprised and distressed; we began to suspect that our eyes deceived us; we looked again, and again; but, alas! there was no mistake; we plunged from one step of bewilderment to another still more profound, until at length we were utterly lost in stark, if not indignant, astonishment. Before giving our readers the benefit of a few extracts from Mr. Newnham's work, it may be as well to inform them that, not more than twelve months ago, he was so little of an admirer of Animal Magnetism that, he was *then* actually "asked by some friends to write a paper against it;" and that *now* he is so undoubting and devoted a believer in its whole truth, that he readily gives his entire assent to all the mysteries of Clairvoyance, Translation of the Senses, Foreknowledge of Events, &c.; and is moreover so smitten with the anticipations of the value of his own performances, that he addresses his friend (to whom he dedicates the work) as one "to whom I think posterity will be grateful for having stimulated an enquiry, which I venture to foresee will ultimately be productive of relief to some of the sorrows of life." Now for a few illustrations of this enquiry that is so rich in promise of good to mankind. The following is a *fact*.

"During clairvoyance, *no knowledge is elicited*, no opinion given, *not previously possessed* by the magnetiser or magnetised,—at least in its germ, if not in its development. For instance, a Turkish somnambulist will never give *christian views or opinions, unless the magnetiser be a Christian*." 200.

A little farther on we read that—

"Somnambulists may be more or less clairvoyans,—more or less perfect, —and may offer very varying phenomena: but the almost invariable attributes of this state are the faculties of seeing with their eyes closed,—their intimate connexion with their magnetiser,—the development of their intellectual faculties,—the insight into their own structure,—and the foresight of their approaching maladies." 237.

The following piece of metaphysical transcendentalism we must give without comment, for the best of all reasons; we do not understand it.

"We believe in the existence of sight, without the aid of the eyes in the magnetic condition;—but the measure of this belief is not the same as that which we possess in consequence of seeing with our own eyes. And the reason is obvious. First, if we doubt about the report of our eyes, and before lengthened habit has taught us to trust in their accuracy, we test that accuracy by the aid of some of the other senses, as touch, taste, &c.:—witness the attainment of knowledge by infants: and clairvoyance should be considered somewhat in the same light. Then, again, long habit has taught us to trust in the report of the eyes;—and for the same reason, we SEEM *to understand* that report: while, on the other hand, though we admit the facts, they do so manifestly transcend our present understanding, that although not *more inexplicable* than ordinary sight, *sixty years after the creation*, now *seem* to be so, on account of the difference of associated circumstances." 293.

Whether it be from diffidence, or from the hitherto-limited sphere of his experience in the mysteries of his new craft, that Mr. Newnham adduces so few cases from his own note-book, we cannot tell; but certain it is, that almost all his reports of cases are derived from foreign works. The former are, as a matter of course, doubly valuable, and deserve our special notice upon the present occasion. What do our readers think of this one?

After giving the particulars of "an exceedingly good case" of epilepsy in a girl 11 years of age, cured mesmerically by Dr. Inglis of Halifax, (who very modestly remarks at the close of his report that "an approving conscience gave me my reward,") Mr. Newnham proceeds to observe:—

"A similar case reached me a few days since, from a clergyman:—and it is not many weeks since a lady was describing to me the agonies of her baby, and its cries for three hours, which nothing would pacify till she *stripped it and herself,* and laid it upon her stomach, when in a few minutes it was relieved, and sank into a peaceful slumber. Here again was a case of unconscious magnetism —and no possibility of the intervention of imagination." 141.

The next case of our author's, that we have been able to ferret out from amidst a farrago of the most astounding trash, is the following.

A young clergyman mesmerised his sister, who soon fell asleep, and continued so for five hours, in spite of all his endeavours to awake her. After distinctly exhibiting some of the phenomena of Clairvoyance, &c., he wished to arouse her; but this he could not do with all his endeavours. He at last bethought himself of asking her *whether she would awake naturally, and when*—to which she replied affirmatively, and *in half an hour:* this took place accordingly, and exactly.

This remarkable "case of genuine unsophisticated magnetism with clairvoyance and prevision," we are informed, "has recently come within our (author's) own knowledge."

His third case reminds us much of the far-famed one of the Okeys; and who is there that has not heard of Dr. Elliotson's wonderful girls? By-the-bye, this prince of English Mesmerists is rather shabbily treated, we think, by one who is but a neophyte and sciolist in the art. At page 124 of his work, Mr. Newnham very discourteously remarks, that "the doctor is no friend or favourite of ours;" and subsequently, when discussing the metaphysico-physical subject of Phreno-Magnetism, he thus describes the character of his mind.

"We must allow," says he, "that this state, if it be true, is a very extraordinary one; and we may even earnestly wish that it had been introduced to notice by persons of less lively imagination, and more sober judgment than Drs. Elliotson and Engledue:—for, although we entertain the highest respect for their talents, we cannot say that we equally highly appreciate their judgment; and we confess that we think their imaginations too liable to be captivated by the charms of novelty,—and therefore more easily led astray by some brilliant and unreal phantom." 376.

He afterwards compensates in some degree for this niggard praise, by styling Drs. Elliotson and Engledue, "these two great men, *whose cerebral organism seems to have been cast into* (the Italics are not ours) the same mould:—pity is it, that we are not acquainted with the cerebral organisms of their immediate progenitors!"

Mr. Newnham, we should remark, eloquently defends his newly-adopted science from the charge of ignoble birth and short descent. It was known, he tells us, in the remotest times, to the Hindoos and Egyptians, the Jews and Persians. By the Greeks, it was termed the *sacred mysteries;* and in the middle ages it was known under the names of *magic,* the *black art,* and the *occult sciences,* &c. He takes vast pains to prove that it is one of the greatest boons, which Heaven in its mercy has bestowed upon mankind. His zeal, however, rather than his discretion, is manifested by the constant introduction of religious topics and language that he indulges in. But on this, as on most other matters, his judgment cannot be well trusted. For example, he very unnecessarily proclaims himself to be a Materialist. In excuse it may be said, that he sometimes plunges into the discussion of subjects that are far too abstruse for his mind; and then he gets bewildered, and gives utterance to what he does not fully understand.* Even on mere professsional matters, his opinions seem to be often most unsteady and unsound;—so much so, that we have more than once, during its perusal, hesitated to believe that this work could have been written by an educated medical man. Neither can its literary merits be estimated much higher. The sentences sometimes extend to a page or two in length, and are every now and then garnished with the merest *niaiserie* of thought and language. We looked for *facts*, and our author gives us *words*. Among other things he supplies a sketch of Chardel's views, the foundation of which is, that *light is the principle of life*. What they have to do with the subject-matter of his book, it is hard to comprehend. He re-produces the long reports of the French Academy on the subject of animal magnetism; betraying, every now and then, a most unpardonable ignorance—we shall not say a discreditable misrepresentation—of the characters of some of the ablest men in the French metropolis. He repeats, and seemingly with credulous approbation, the ravings of a madman before the French Revolution, and the "remarkable previsions" of poor Joan of Arc! The existence of the *second sight* of the Scottish Highlanders is also put down as an undoubted fact! But what can we expect from a man who avows his belief that water and food and trees may be magnetised for the benefit of man! "Tell it not in Gath, nor publish it in the streets of Ascalon;" else every *quack* of the realm will start into madness, and, joining together as with magnetic unanimity, will cry aloud for their rights, and call on Sir James Graham to declare, how far the *regulars* may go, and where empiricism is to begin.

Our readers have probably had quite enough of Mr. Newnham by this time. If any one should ask why we have been so severe in our comments on his work, we answer at once that we have had a twofold reason: first, the silly character of its contents; and secondly, his unwarrantable aspersion of the motives of his professional brethren who do not think as he does. Passing over his remarks on the incompetency and want of candour of medical men in examining such an abstruse subject as that of Animal Magnetism, we must look back to page 15, where he does not hesitate to charge them with mercenary considerations in their opposition to its claims:—

"We shall mention but one other cause, and we trust not *very* operative, but still *one*, which to a *certain extent* must have its influence. 'Il faut vivre,'—and true, most true it is, that in general it is very sorry living; and that medical men are engaged in a perpetual and most arduous struggle, to maintain themselves and their families in their proper station. Now, in spite of the most disinterested benevolence, the law of self-preservation will be uppermost, and there must be an inevitable prejudice excited against every thing which may decrease the means

---

* What other explanation can be given of his inconsistency? for, at the commencement of his work, we meet with the following passage:

"Thus magnetism has been combined with scepticism and infidelity, and it is not to be denied that it has been often thus *associated*. But it has no *necessary* connexion with such errors; and the *conversion of Georget* late in life from materialism to spiritualism, is entirely to be attributed to his conviction of the truth of magnetic phenomena." 5.

of subsistence,—and of this number must be reckoned human magnetism ; but if so, its friends will be slowly gained, and generally recruited from those who are not so dependent for their daily food. It is impossible not to admit, that this argumentum ad crumenam *must* bias the minds of many against any change which may endanger the hope of their gains, and tend in any degree to supersede their craft, or at all events to endanger its estimation." 16.

Has the writer of such a passage as this any right to look for leniency at the hands of those who seek to uphold the dignity of our profession ?

With respect to Mr. Greenhow's Report of Miss Martineau's case—which, by the bye, seems to be thought highly of by Mr. Newnham—we should have thought that it did not merit the distinction of being made the subject of a separate pamphlet. The lady seems to have suffered at first from a sort of chronic inflammation or irritative condition of the uterus. This organ subsequently became retroverted, and swollen in its substance. Sir Charles Clarke was consulted in 1841, and evidently formed a favourable opinion as to the *result* of the affection. As a matter of course, there had been all along more or less constitutional disturbance, and especially of the nervous and digestive systems. The general health had for some time been improving, and the local malady diminishing, under the judicious treatment that had been followed out under the directions of Mr. Greenhow ; when, in June last, she commenced the Mesmeric treatment—which has worked such marvels, according to her own report. Any other novel and pleasing impression on her mind would doubtless have done the same. Dare we hint at the probable effect which a change of name might have had, even upon a lady of her Malthusian principles ? Be this as it may, we venture to predict that the permanency of her cure will depend very much upon herself. If she loses faith in Magnetism, and does not substitute some other pleasing whim in its stead, it would surprise us little to hear of her falling back into her former state of health—when, says Mr. Greenhow, " she never willingly listened to my suggestions of the probability of such prospective events (her recovery, &c.) and seemed always best satisfied with anything approaching to an admission that she must ever remain a secluded invalid." This, by-the-bye, is a curious, but not uncommon feature of certain states of *ill health* in unmarried women.

If Mr. Pyne is not a better priest than he is a physician, woe betide his parishioners : they must surely have good cause to repine. Among other curiosities, the Rev. gentleman is of opinion that Naaman the leper, of whom we read in Scripture, certainly alluded to animal Magnetism, when he said of Elisha : " I thought he would surely come out to me, and stand and call on the name of the Lord his God, and strike his hand over the place (move his hand up and down—Hebrew), and recover the leper."

Some of Mr. Pyne's own cures seem to us to be nearly as miraculous as that effected by the Prophet himself. For example : A man had a rusty edging iron run into his foot ; a surgeon told him it had gone to the bone ; he was in great pain, and unable to put his foot to the ground. He was mesmerised ; in a few minutes he was well, and signed his name to these words :—" I feel cured, I thank God. I shall carry my crutch and stick, for I shall run home, or a part of the way." Who dare resist the claims of Animal Magnetism after this ? Scoffers and infidels, beware !

